# Socioeconomic inequalities in occupational, leisure-time, and transport related physical activity among European adults: A systematic review

**DOI:** 10.1186/1479-5868-9-116

**Published:** 2012-09-19

**Authors:** Marielle A Beenackers, Carlijn BM Kamphuis, Katrina Giskes, Johannes Brug, Anton E Kunst, Alex Burdorf, Frank J van Lenthe

**Affiliations:** 1 Department of Public Health, Erasmus MC, Rotterdam, Netherlands; 2 School of Public Health/Institute for Health and Biomedical Innovation, Queensland University of Technology, Brisbane, Australia; 3 School of Medicine, University of Sydney, Sydney, Australia; 4 Department of Epidemiology & Biostatistics and the EMGO Institute for Health and Care Research, VU University Medical Centre, Amsterdam, Netherlands; 5 Department of Public Health, AMC, University of Amsterdam, Amsterdam, Netherlands

**Keywords:** Socioeconomic, Inequalities, Physical activity, Systematic review, Europe

## Abstract

**Background:**

This study systematically reviewed the evidence pertaining to socioeconomic inequalities in different domains of physical activity (PA) by European region.

**Methods:**

Studies conducted between January 2000 and December 2010 were identified by a systematic search in Pubmed, Embase, Web of Science, Psychinfo, Sportdiscus, Sociological Abstracts, and Social Service Abstracts. English-language peer-reviewed studies undertaken in the general population of adults (18–65 years) were classified by domain of PA (total, leisure-time including sport, occupational, active transport), indicator of socioeconomic position (education, income, occupation), and European region. Distributions of reported positive, negative, and null associations were evaluated.

**Results:**

A total of 131 studies met the inclusion criteria. Most studies were conducted in Scandinavia (n = 47). Leisure-time PA was the most frequently studied PA outcome (n = 112). Considerable differences in the direction of inequalities were seen for the different domains of PA. Most studies reported that those with high socioeconomic position were more physically active during leisure-time compared to those with low socioeconomic position (68% positive associations for total leisure-time PA, 76% for vigorous leisure-time PA). Occupational PA was more prevalent among the lower socioeconomic groups (63% negative associations). Socioeconomic differences in total PA and active transport PA did not show a consistent pattern (40% and 38% positive associations respectively). Some inequalities differed by European region or socioeconomic indicator, however these differences were not very pronounced.

**Conclusions:**

The direction of socioeconomic inequalities in PA in Europe differed considerably by domain of PA. The contradictory results for total PA may partly be explained by contrasting socioeconomic patterns for leisure-time PA and occupational PA.

## Introduction

Socioeconomic inequalities in morbidity and mortality are well-documented [1,2]. Differences in health behaviours play an important role in these inequalities [3]. Next to the higher prevalence of smoking in lower socio-economic groups [4,5], evidence suggests that the higher obesity rates are of major importance to health inequalities [6-9].

Obesity levels in Europe are rising rapidly; the prevalence of obesity has tripled since the 1980s [10]. This high prevalence of obesity is estimated to account for 1 million deaths and 12 million life years of ill health in Europe each year [10]. European regions are thought to be in a different stage of the obesity epidemic; when the level of economic development increases, the proportion of positive associations between socioeconomic position (SEP) and overweight and obesity decreases and the proportion of negative association increases [6,7]. Because overweight and obesity are the result of an excessive energy intake or limited energy expenditure, differences in dietary intake or physical activity (PA) are expected to contribute to the socioeconomic inequalities in overweight and obesity. A recent review of socioeconomic inequalities in nutrition in Europe [11] reported that consistent socioeconomic inequalities in diet were seen for fruit and vegetable consumption and, to a lesser degree, for fibre consumption but not in amounts of energy intake. PA is a health behaviour of major importance as it is strongly associated with obesity and a number of diseases such as metabolic disease and certain cancers [12,13]. However, no systematic review of the evidence of socio-economic differences in PA in Europe has been published to date.

PA is often categorized as low intensity PA (<3 Metabolic Equivalent (MET)) versus moderate (3–6 METs) to vigorous PA (>6 METs) [14]. The latter two categories are regarded as especially important for health. Furthermore, leisure-time, work-related, and transport-related PA are often distinguished from each other. Empirical evidence suggests that socioeconomic patterns may differ for different domains of PA [15,16]. Patterns may also differ by gender, as exemplified by the finding that inequalities in overweight and obesity are larger in women [7], and by European region, as illustrated by the North to South gradient in obesity inequalities [6,7]. Finally, traditional indicators of SEP, such as income, occupation, and education, may reflect different aspects of one’s position in the social stratification [17,18], and may therefore be more strongly or weakly related to specific outcomes.

The purpose of this review is to describe socioeconomic inequalities in different domains of physical activity, across different SEP indicators, in men and women, and across different regions in Europe.

## Method

### Search strategy

#### Databases and search terms

Major databases (PubMed, EMbase, Web of Science, PsychINFO, SportDiscus, and Sociological Abstracts and Social Services Abstracts) were searched to locate relevant studies published between the first of January 2000 and the 31^st^ of December 2010. Broad search terms, including synonyms, were used to ensure that all potentially relevant articles were included in the search results. When possible, database specific search terms were used to optimize the results. The search strategy and syntax for each database are available from the authors (MAB) upon request.

#### Inclusion and exclusion criteria

Publications were included if they were published in English-written peer-reviewed journals. Studies had to be conducted among the general population, which therefore excluded studies utilizing patient groups. Given the interest in occupational physical activity, study participants had to be of working age (18–65 years of age). Studies quantitatively assessed the association between at least one SEP indicator and one domain of physical activity (measured, either in terms of frequency (e.g. times/week), duration (e.g. hours or minutes), and/or intensity (e.g. vigorous)). Indicators of SEP included education, social class (based on occupation), income (either individual or household level), household wealth (e.g. car ownership, housing tenure) or area-based indicators (e.g. area deprivation). Outcomes included were total physical activity, leisure-time physical activity including but not limited to sports and exercise (both organized and unorganized), active transport (walking, cycling), and occupational physical activity. Manuscripts that elicited concerns about the study quality were excluded. These quality concerns were inconsistencies between the results in the text and the results in the tables, missing information on how the outcome or SEP indicator was measured, or missing information on the basic description of the sample, such as sample size.

### Data extraction and summarization

#### Title scanning and selection

As a first step in identifying relevant studies, titles and abstracts were read by the lead author (MAB). Second, the full text was read if studies met the inclusion criteria and when it was clear from the title and/or abstract that the association between SEP and PA was studied. A second researcher (CBMK) executed an independent parallel selection process with a random subsample of 200 titles and abstracts which resulted in a similar selection.

#### Data synthesis

The following information was extracted into data extraction tables from each included study: country, year (or years) the data were collected, sample size and sample characteristics (in case a subpopulation was studied), age range, percentage males, percentage response, SEP indicator and PA outcomes (Table 1).

**Table 1:** Characteristics of the 131 included studies ordered by European region

**Author, year of publication**	**Country of study**^**a**^	**Study name**	**Year of data- collection**	**Sample size + characteristic**^**b**^	**Age**	**% Male**^**c**^	**Response**^**c**^	**SEP indicator**^**d**^	**PA domain**^**e**^
**EU wide studies**									
Martinez-Gonzales *et al.*, 2001 [54]	EU – 15 countries	Pan-European Union survey	1997	n= 15239	15+	47%	NP	Education	TLTPA
Ståhl *et al.*, 2001 [60]	BEL, FIN, DEU, NLD, ESP, SWI	MAREPS project	1997-98	n= 3343	18+	43%	54%	Education	TPA
Van Tuyckom & Scheerder, 2008 [61]	EU – 27 countries	Eurobarometer 64.3	2005	n= 26688	15+	NP	NP	Education Social class	TLTPA
Van Tuyckom & Scheerder, 2010a [62]	EU – 27 countries	Eurobarometer 64.3	2005	n= 26362	15+	NP	NP	Social class	TLTPA
Van Tuyckom & Scheerder, 2010b [55]	EU – 27 countries	Eurobarometer 64.3	2005	n= 26688	15+	NP	NP	Education	OPA TLTPA AT
Varo *et al.*, 2003 [56]	EU – 15 countries	Pan-European Union survey	1997	n= 15239	15+	47%	NP	Education	TLTPA
**Western European region**									
Addor *et al.*, 2003 [63]	SWI	Health examination survey of adults (MONICA project)	1992-93	n= 1550	25-64	49%	53%	Education	VLTPA
Bertrais *et al.*, 2004 [64]	FRA	SUVIMAX study	1998	n= 7404	45-68	46%	NP	Education	TLTPA
Chaix & Chauvin, 2003 [65]	FRA	2000 French Health Monitoring Survey	2000	n= 12948	16+	49%	66%	Education Income	TLTPA
Dragano *et al.*, 2007 [66]	DEU, CZE	DEU: Heinz Nixdorf Recall (HNR) Study CZE: Health, Alcohol & Psychosocial Factors in Eastern Europe (HAPIEE)	DEU: 2000-03	n DEU = 4032	45-69	DEU: 49%	DEU: 56%	Education Neighb. SEP	TLTPA
			CZE: 2002-05	n CZE = 7522		CZE: 45%	CZE: 55%		
Drieskens *et al.*, 2010 [67]	BEL	Belgian Health Interview Survey (HIS)	1997	n 1997 = 7431	15+	NP	60%	Education	TLTPA
			2001	n 2001 = 8142					
			2004	n 2004 = 7459					
Galobardes *et al.*, 2003 [68]	SWI	Bus Santé 1993-2000	1993-2000	n= 8194	35-74	51%	57-65%	Education Social class	VLTPA
Kamphuis *et al.*, 2008 [52]	NLD	Dutch GLOBE study 2004	2004	n= 3839	25-75	48%	64%	Income Education	VLTPA
Kamphuis *et al.*, 2009 [51]	NLD	Dutch GLOBE study 2004	2004	n= 1994	55-75	48%	62%	Income Education	TLTPA
van Lenthe *et al.*, 2005 [47]	NLD	Dutch GLOBE study 1991	1991	n= 8767	20-69	NP	70%	Neighb. SEP	TLTPA VLTPA AT
Meyer *et al.*, 2005 [69]	SWI	Swiss Health Survey 2002	2002	n= 8405	50+	45%	NP	Income Education	VLTPA
				community residents					
Nocon *et al.*, 2008 [70]	DEU	German National Health Survey	1998	n= 7124	18-79	48%	NP	Income Education Social class	VLTPA
van Oort *et al.*, 2004 [71]	NLD	Dutch GLOBE study 1991	1991	n= 16980	15-74	49%	70%	Education	TLTPA
Rathmann *et al.*, 2005 [72]	DEU	KORA (=Cooperative Health Research in the Region of Augsburg) Survey	2000	n= 1653	55-74	51%	62%	Income Education Social class	TLTPA
Ribet *et al.*, 2001 [73]	FRA	GAZEL study (G) MONICA – France (M)	G: 1989-92 M: 1994-97	n G = 9486 n M = 534 working, living in couple	40-50	100%	G: 44-87% M: 51-77%	Social class	TLTPA
Scheerder *et al.*, 2002 [74]	BEL	Sports participation in Flanders	1969 1979 1989 1999	n 1969 = 7479	NP	50%	71-89%	Education Social class	VLTPA
		- Leuven Growth Study of Flemish Girls		n 1979 = 18629					
		- Study on Movement Activities in Flanders		n 1989 = 7957					
				n 1999 = 9143					
				parents of school children					
Scheerder *et al.*, 2005 [75]	BEL	Sports participation in Flanders	1979 1989 1999	n 1979 = 19396	NP	50%	71-89%	Education Social class	VLTPA
		- Leuven Growth Study of Flemish Girls		n 1989 = 8624					
		- Study on Movement Activities in Flanders		n 1999 = 10356					
				parents of school children					
Schneider & Becker, 2005 [76]	DEU	German National Health Survey	1998	n= 3323 employed	18-69	56%	61%	Income Education Social class Individual SEP	VLTPA
Van Dyck *et al.*, 2010 [23]	BEL	Belgian Environmental Physical Activity Study (BEPAS)	2007-08	n= 1166	20-65	48%	58%	Education Neighb. SEP	TLTPA AT
Verdaet *et al.*, 2004 [77]	BEL	BELSTRESS study (subsample)	NP	n= 892 working men	35-59	100%	NP	Education	TLTPA
de Vries *et al.*, 2008 [78]	NLD	SMILE study	2002	n= 9449	12+	42%	NP	Education	TPA
Wagner *et al.*, 2003 [79]	FRA IRE	PRIME Study – France	NP	n FRA = 7359	50-59	100%	NP	Education Household wealth	TLTPA VLTPA AT
		PRIME Study – Ireland		n IRE = 2398					
**Eastern European region**									
Drygas *et al.*, 2009 [31]	POL	National Polish Health Survey, (WOBASZ, Project)	2002-05	n= 12552	20-74	47%	74-79%	Income Education	TLTPA
Frömel *et al.*, 2009 [80]	CZE	Czech physical activity, environment and SES study	NP	n= 9950	25-64	49%	58%	Individual SEP	TPA VLTPA
Jurakić *et al.*, 2009 [81]	HRZ	Croatian physical activity study	2007	n= 1032	15+	48%	NP	Income Education	TPA OPA TLTPA AT
Kaleta & Jegier, 2005 [32]	POL	Physical activity in Poland	NP	n= 508 employed	adults (42 ± 10)	54%	47%	Income Education	TLTPA
Kaleta & Jegier, 2007 [82]	POL	Physical activity in Poland	NP	n= 954	25-64	47%	48%	Income Education	TLTPA
Kwaśniewska *et al.*, 2010 [28]	POL	The National Multicentre Health Survey (WOBASZ Project)	2004-05	n= 7280 works/ studies outside home	20-74	51%	74-79%	Income Education	AT
Leskošek *et al.*, 2002 [83]	SVN	Sport participation in the Republic of Slovenia	1998	n= 1768	18+	52%	59%	Education	VLTPA
Mišigoj-Durakoviæ *et al.*, 2000 [84]	HRZ	Zagreb study	1999	n= 594 employed	20-65	50%	20%	Education	OPA TLTPA VLTPA
Nowak, 2010 [85]	POL	Western Poland active lifestyle survey	2000-06	n= 3662	20-75	all female	NP	Education	VLTPA
Paulik *et al.*, 2010 [86]	HUN	Health survey rural Hungary	2006	n= 3380 living in small settlements	18+	47%	83%	Education Household wealth	VLTPA
Pomerleau *et al.*, 2000 [87]	EST LVA LTU	Three national surveys of adults	1997	n EST = 2018	19-65	EST: 45%	EST: 67%	Income Education	TLTPA VLTPA
				n LVA =		LVA:	LVA:		
				2303		46%	78%		
				n LTU = 2140		LTU: 44%	LTU: 73%		
Puska *et al.*, 2003 [88]	EST LTU FIN	Finbalt project	1994, 1996, 1998	n EST = 3808	20-64	EST: 44%	EST: 68-83%	Education	TLTPA
				n LTU = 5716		LTU: 44%	LTU: 62-69%		
				n FIN = 9608		FIN: 48%	FIN: 70-72%		
Shapo *et al.*, 2004 [89]	ALB	Health behaviours and health status in Tirana City	2001	n= 1120	25+	48%	73%	Income Education	TLTPA
Stelmach *et al.*, 2004 [90]	POL	CINDI programme (Countrywide Integrated Noncommunicable Disease Intervention Programme)	2001-02	n= 1837	18-64	54%	NP	Income Education	TLTPA
Zaletel-Kragelj *et al.*, 2006 [91]	SVN	CINDI Health Monitor	2001	n= 7718 without disability	25-64	47%	64%	Education Social class	TPA
**Southern European region**									
Artazcoz *et al.*, 2004 [92]	ESP	Catalonian Health Survey (CHS)	1994	n= 2866 workers and housewives	25-64	all female	NP	Education	VLTPA
Bolívar *et al.*, 2010 [93]	ESP	Andalusia Health Survey	1999, 2003	n= 13193	16+	49%	NP	Education Social class Neighb. SEP	TLTPA
Borrell *et al.*, 2000a [94]	ESP	Barcelona Health Interview Survey	1992	n= 4171	14+	47%	91%	Occupation	TPA VLTPA
Borrell *et al.*, 2000b [95]	ESP	Barcelona Health Interview Survey	1986	n 1986 = 7907	14+	1986: 46%	88-93%	Occupation	TPA
			1992	n 1992 = 5004		1992: 47%			
			1994	n 1994 = 2155		1994: 44%			
De Vogli *et al.*, 2005 [96]	ITA	Health Determinants Surveillance System (HDSS) Survey	2003	n= 3327	18-91	52%	57%	Social class	TLTPA
Gal *et al.*, 2005 [97]	PRT	Porto health survey	NP	n= 2004	18+	39%	70%	Education Social class	TPA TLTPA
Lera-López & Rapún- Gárate, 2005 [98]	ESP	Sport participation and consumer expenditure in Navarra, Spain	2004	n= 700	16-65	NP	NP	Income Education	VLTPA
Meseguer *et al.*, 2009 [99]	ESP	Non-communicable Disease Risk Factor Surveillance System (NCDRFSS)	2000-05	n= 12037	18-64	49%	65%	Education	TLTPA
Panagiotakos *et al.*, 2008a [100]	GRC	ATTICA study	2001-02	n= 3042	18+	50%	75%	Education	TPA
Panagiotakos *et al.*, 2008b [101]	GRC	ATTICA study	2001-02	n= 3042	18+	50%	75%	Education	TPA
Pascual *et al.*, 2007 [102]	ESP	Spanish Health Study	2001	n= 19324	16-74	49%	85%	Income Education Social class Neighb. SEP	TLTPA
Pascual *et al.*, 2009 [103]	ESP	General survey on customs regarding media and leisure activities	1999	n= 25982	25-74	49%	70%	Income Education Neighb. SEP	VLTPA
Pitsavos *et al.*, 2005 [104]	GRC	ATTICA study	2001-02	n= 3042	20-89	50%	75%	Income Education Social class	VLTPA
Santos *et al.*, 2009 [105]	PRT	Azorean Physical Activity and Health Study	2004	n= 9991	18-65	43%	88%	Income Education	TPA
Schröder *et al.*, 2004 [106]	ESP	Gerona cardiovascular risk factor and lifestyle study	1994-96	n= 1748	25-74	48%	73%	Education	TLTPA VLTPA
**Scandinavian region**									
Ali & Lindström, 2006 [107]	SWE	2000 public health survey in Scania	2000	n= 5180 workforce or unemployed	18-64	56%	59%	Education	TLTPA
Andersen *et al.*, 2000 [108]	DNK	Copenhagen City Heart Study (CCHS)	1964-92	n= 30640	20-93	56%	69-95%	Education	TLTPA VLTPA AT
		Copenhagen Male Study (CMS)							
		Glostrup Population Study (GPS) (pooled)							
Barengo *et al.*, 2006 [109]	FIN	National FINRISK Study	1972-97	n= 33712	30-59	49%	71-95%	Education Social class	TPA
Bergman *et al.*, 2008 [110]	SWE	International Prevalence Study (IPS) Sweden	2003	n= 1470	18-74	47%	59%	Income Education	TPA
Borodulin *et al.*, 2008 [111]	FIN	National FINRISK Study	2002	n= 4437	25-64	44%	59-70%	Education	TLTPA
Cubbin *et al.*, 2006 [112]	SWE	Swedish Annual Level of Living Survey	1996-2000	n= 10890	25-64	49%	80%	Individual SEP Neighb. SEP	VLTPA
Engström, 2008 [113]	SWE	Sport Habitus Study Sweden	2007	n= 1518	53	NP	77%	Education	VLTPA
Häkkinen *et al.*, 2006 [114]	FIN	Northern Finland 1966 Birth Cohort	1998	n= 4343	31	46%	76%	Education	TLTPA
Henriksson *et al.*, 2003 [115]	SWE	Cardiovascular Risk Factor Study in Southern Sweden (CRISS)	1990	n 1990 =	37	100%	1990:	Education	TLTPA
			1993	991	40		68%		
			1996	n 1993 = 770	43		1993: 78% *		
				n 1996 = 702			1996: 71% *		
							*of baseline		
Hu *et al.*, 200 [37]	FIN	National FINRISK Study	1982, 1987, 1992	n= 14290	35-64	48%	74-88%	Education	OPA TLTPA AT
Kivimäki *et al.*, 2007 [116]	FIN	Finnish Public Sector Study	2000-02	n= 48592	17-65	19%	68%	Individual SEP	TPA
Korniloff *et al.*, 2010 [117]	FIN	Finnish type 2 diabetes (FIN-D2D) survey	2007	n= 2778	45–74	47%	64%	Income Education	TLTPA
Laaksonen *et al.*, 2002 [118]	FIN	Finnish Adult Health Behaviour Survey	1991-98	n= 26014 civil servants	15-64	47%	69-76%	Education	TLTPA
Laaksonen *et al.*, 2008 [119]	FIN	Finnish Adult Health Behaviour Survey	1979-2001	n= 60608	25-64	48%	62-86%	Education	TLTPA
Lagerros *et al.*, 2009 [120]	SWE	The Swedish National March Cohort	1997	n= 42150	18-94	36%	NP	Education	TPA
Leijon *et al.*, 2010 [121]	SWE	Public Health Survey Ostergotland County	2006	n= 6966	18-84	45%	54%	Education Self-reported economy	TPA
Lindström *et al.*, 2001 [122]	SWE	The Malmö Diet and Cancer Study	1992-94	n= 11837	45-65	45%	39%	Social class	TLTPA
Lindström *et al.*, 2003a [123]	SWE	The Malmö Public Health Survey	1986, 1994	n= 3861	21-81	47%	71-74%	Education	TLTPA
Lindström *et al.*, 2003b [124]	SWE	The Malmö Public Health Survey	1994	n= 3377	20-80	NP	71%	Education	TLTPA
Mäkinen *et al.*, 2009 [125]	FIN	Finnish Adult Health Behaviour Survey	1978-2002	n= 50815 employed	25-64	50%	62-86%	Income Education Social class	TLTPA AT
Mäkinen *et al.*, 2010a [126]	FIN	The Health 2000 Survey	2000-01	n=3355 employed	30+	46%	85-89%	Social class	OPA TLTPA
Mäkinen *et al.*, 2010b [127]	FIN	The Health 2000 Survey	2000-01	n= 7112	30+	45%	84-89%	Income Education Social class	TLTPA
Mäkinen *et al.*, 2010c [128]	FIN	National FINRISK study	2002	n= 4408	25-64	44%	60-70%	Education	TLTPA
Molarius, 2003 [129]	SWE	Varmland County Survey	2000	n= 6394	25-74	47%	70%	Education	TLTPA
Nielsen *et al.*, 2006 [130]	DNK	Odense Androgen Study	2002, 2003	n= 783	20-29	100%	73%	Education	TLTPA
Norman *et al.*, 2002 [131]	SWE	COSM (cohort of Swedish men)	1997	n= 33466	45-79	100%	48%	Education	TPA TLTPA
Novak *et al.*, 2006 [132]	SWE	Swedish Cohort Study	1981, 1995	n= 1044	16, 30	52%	96%	Education	TPA
Orsini *et al.*, 2007 [133]	SWE	Swedish Mammography Study (SMC97)	1997	n= 38988	40-75	all female	70%	Education	TPA
Osler *et al.*, 2000 [134]	DNK	MONICA – Denmark	1982-1984, 1987, 1991-92	n= 6695	30, 40, 50, 60	50%	73-79%	Education	TLTPA
Osler *et al.*, 2001 [135]	DNK	Children of the Copenhagen City Heart Study	1992	n= 317	19-31	51%	52%	Education	TLTPA
Osler *et al.*, 2008 [136]	DNK	Metropolit cohort (1965)	2004	n= 6292	51	100%	66%	Education	TLTPA
Petersen *et al.*, 2010 [137]	DNK	Danish National Health Interview Survey	1987	n 1987 = 4752	16+	49%	1987: 80%	Education	TLTPA
			1994	n 1994 = 4667			1994: 78%		
			2000	n 2000 = 16688			2000: 74%		
			2005	n 2005 = 14566			2005: 67%		
Piro *et al.*, 2007 [138]	NOR	Oslo Health Study (HUBRO)	2000	n= 14608	30, 40, 45, 60	45%	46%	Income Education Neighb. SEP	VLTPA
Pudaric *et al.*, 2000 [139]	SWE	Migrants in Sweden Study	1988-89	n= 3100	55-74	47%	80%	Income	TPA
Pulkki *et al.*, 2003a [140]	FIN	Cardiovascular Risks in Young Finns (CRYF) study	1983, 1992	n= 1219	12-21, 21-30	44%	62%	Individual SEP	TLTPA
Pulkki *et al.*, 2003b [141]	FIN	Cardiovascular Risks in Young Finns (CRYF) study	1983, 1992	n= 1125	12-21, 21-30	58%	57%	Education	TLTPA
Salonen *et al.*, 2010 [142]	FIN	Sub-study of the Helsinki Birth Cohort Study	2001-04	n= 1967	57-71	46%	NP	Education Social class	TLTPA
Schnohr *et al.*, 2004 [143]	DNK	Copenhagen City Heart Study (CCHS)	1967-86	n= 30635	20-93	53%	NP	Education	TLTPA
		Copenhagen Male Study (CMS)							
		Glostrup Population Study (GPS) (pooled)							
Simonen *et al.*, 2003 [144]	FIN	Finnish Twin Cohort	1975, 1981	n= 224 monozygotic twins	35-69	NP	82%	Education	VLTPA
Sjögren & Stjernberg, 2010 [145]	SWE	Swedish National Study on Aging and Care (SNAC)	2001-03	n= 999	60-96	45%	61%	Education	TLTPA
Strand & Tverdal, 2004 [146]	NOR	Cardiovascular disease study in Norway	1970	n= 44684	35-49	51%	91%	Education	TLTPA
Strandhagen *et al.*, 2010 [147]	SWE	The INTERGENE research programme	2001-04	n= 3581	25-74	47%	42%	Education	TLTPA
Suadicani *et al.*, 2001 [42]	DNK	Copenhagen Male Study	1970-71	n= 5028	40-59	100%	87%	Social class	OPA TLTPA
Suadicani *et al.*, 2005 [148]	DNK	Copenhagen Male Study	1970-71 1985-86	n= 3290	40-74	100%	75-87%	Social class	TLTPA
Tammelin *et al.*, 2003 [149]	FIN	Northern Finland 1966 Birth Cohort	1998	n= 7794	31	46%	75%	Education	TLTPA
Wang *et al.*, 2010 [34]	FIN	National FINRISK Study (pooled data)	1972, 1977, 1982, 1987, 1992, 1997, 2002	n= 58208	24-74	49%	65-88%	Education	OPA TLTPA AT
Wemme & Rosvall, 2005 [150]	SWE	Scania Health Survey	1999-2000	n= 7169 employed	NP	54%	59%	Education Social class	TLTPA
**Anglo-Saxon region**									
Adams, 2009 [151]	GBR	English Longitudinal Study of Ageing (ELSA)	2002	n= 10864	50+	47%	NP	Education	TPA
Adams, 2010 [29]	GBR	2005 UK Time Use Survey (part of National Statistics Omnibus Survey)	2005	n= 3933	16+	48%	49%	Education Social class	AT
Allender *et al.*, 2008 [15]	GBR	Health Survey for England	2003	n= 13974	16+	45%	66%	Education Social class	TPA TLTPA
Amuzu *et al.*, 2009 [152]	GBR	British Women’s Heart and Health Study	1999-2001	n= 3522	60-79	all female	NP	Individual SEP Neighb. SEP	TPA
Bartley *et al.*, 2000 [153]	GBR	Health and Lifestyle study (HALS)	1984	n 1984 = 2176	20-64	100%	NP	Social class	VLTPA
		Health Survey for England (HSfE)	1993	n 1993 = 4723					
Bartley *et al.*, 2004 [154]	GBR	Whitehall II Study	1985-88	n= 5458 civil servants	35-55	74%	73%	Social class	TLTPA
Chaudhury & Shelton, 2010 [155]	GBR	Health Survey for England (HSfE)	2006	n= 1550	60-69	46%	NP	Income Social class Neighb. SEP	TPA
Ecob & Macintyre, 2000 [156]	GBR	West of Scotland 20–07 Study	1987, 1988	n= 3036	15, 35, 55	NP	NP	Neighb. SEP	VLTPA
Harrison *et al.*, 2006 [157]	GBR	Physical activity in North-West England	2001	n= 15465	18+	45%	70%	Neighb. SEP Home owner	TPA
Heslop *et al.*, 2001 [158]	GBR	Cohort of workers recruited from workplaces in Western Scotland between 1970 and 1973	1970-73	n= 958 employed	working age	all female	70%	Education Social class Neighb. SEP	TLTPA
Hillsdon *et al.*, 2008 [159]	GBR	British Women's Heart and Health Study	1999-2001	n= 4286	60-79	all female	NP	Individual SEP Neighb. SEP	TPA
Lahelma *et al.*, 2010 [160]	GBR FIN	The London-based Whitehall II study (WHII)	WHII: 1997-99	n WHII= 2678	WHII: 45–	WHII: 76% HHS:	WHII: 73% HHS:	Social class	TLTPA
							67%		
		The Helsinki Health Study (HHS)				17%			
			HHS: 2001-02	n HHS= 8960	60				
				white collar employees	HHS: 40-60				
Livingstone *et al.*, 2001 [161]	IRL	North/South Ireland Food Consumption Survey (NSIFCS)	1997-99	n= 1379	18-64	48%	NP	Social class	VLTPA
Lunn, 2010 [162]	IRL	The Survey of Sport and Physical Exercise	2003	n= 2896	18+	NP	67%	Income Education	VLTPA
Mein *et al.*, 2005 [163]	GBR	Whitehall II study	1997-99	n= 6224	45-69	72%	71%	Social class	TLTPA
				civil servants					
Mullineaux *et al.*, 2001 [164]	GBR	Allied Dunbar National Fitness Survey of English Adults (ADNFS)	1990	n= 2005	16+	NP	NP	Education	TPA
Mutrie & Hannah, 2004 [165]	GBR	West of Scotland Twenty-07 study (3^rd^ wave)	1995-96	n= 2153	24, 44, 64	42%	NP	Social class	OPA TLTPA
Myint *et al.*, 2006 [166]	GBR	EPIC study	1993-97	n= 23085	40-79	46%	NP	Social class	TPA
Poortinga, 2007 [167]	GBR	Health Survey for England	2003	n= 11617	16-64	NP	NP	Social class	OPA VLTPA
Popham & Mitchell, 2006 [168]	GBR	British Household Panel Survey	1996, 1998, 2000, 2002	n= 9473	18-64	48%	74%	Income Education Social class School type (fee-paying)	TLTPA
Popham & Mitchell, 2007 [16]	GBR	2003 Scottish Health Survey (SHS)	2003	n= 5287	25-64	44%	60%	Individual SEP	TPA OPA VLTPA
Popham, 2010 [169]	GBR	2003 Scottish Health Survey (SHS)	2003	n= 2770	35-54	NP	60%	Social class	VLTPA
Stamatakis & Chaudhury, 2008 [170]	GBR	Health Survey for England (HSfE)	1997, 1998, 2003, 2004, 2006	n= 60938	16+	45%	61-71%	Income Education Social class	VLTPA
Stringhini *et al.*, 2010 [3]	GBR	Whitehall II cohort	1985-88	n= 9590	35-55	68%	73%	Social class	TLTPA
				civil servants					
Wardle & Griffith, 2001 [171]	GBR	British Omnibus Study	1999	n= 1790	16+	50%	70%	Social class	VLTPA
Wardle & Steptoe, 2003 [172]	GBR	British Omnibus Study	2000	n= 1691	16+	45%	62%	Social class	VLTPA
Watt *et al.*, 2009 [173]	GBR	British Women’s Heart and Health Study	1999-2001	n= 3523	60-79	all female	NP	Individual SEP	TPA

^a^ EU = European Union, ALB = Albania, BEL = Belgium, CZE = Czech Republic, DEU = Germany , DNK = Denmark, ESP = Spain, EST = Estonia, FIN = Finland, FRA = France, GBR = United Kingdom, GRC = Greece, HRZ = Croatia (local name is Hrvatska), HUN = Hungary, IRL = Ireland, ITA = Italy, LTU = Lithuania, LVA = Latvia, NLD = The Netherlands, NOR = Norway, POL = Poland, PRT = Portugal, SVN = Slovenia, SWE = Sweden, SWI = Switzerland.

^b^ Sample characteristics only provided when a specific subsample from the population was studied (e.g. working people, civil servants, etc.).

^c^ NP = Not Provided.

^d^ SEP = socioeconomic position, Neighb. = neighbourhood, Individual SEP = composite measure of different individual SEP indicators.

^e^ PA = Physical Activity, TPA = Total Physical Activity, OPA = Occupational Physical Activity, TLTPA = Total Leisure-time Physical Activity, VLTPA = Vigorous Leisure-time Physical Activity, AT = Active Transport.

#### Classification of the outcome measures

The following guidelines were used to classify the studies into the different domains of PA:

A PA outcome was categorized as ‘total physical activity’ (TPA) if it concerned a general PA question (not defined whether they mean occupational PA or leisure-time PA) or if the measure included leisure-time PA as well as occupational PA. Total physical activity was often described as ‘usual’ or ‘daily’ physical activity.

A PA outcome was categorized as ‘occupational physical activity’ (OPA) if it was specifically identified as occupational PA in the methods with words such as ‘occupational’ or ‘during work’.

A PA outcome was categorized as ‘total leisure-time physical activity’ (TLTPA) if it was specifically identified as leisure-time PA in the methods with words such as ‘in free time’ or ‘during leisure time’. Exception: leisure-time physical activity that can be defined as vigorous physical activity (see classification criteria below).

A PA outcome was categorized as ‘vigorous leisure-time physical activity’ (VLTPA) if the methods specifically reported that it is about high intensity physical activity, vigorous physical activity, conditioning physical activity, or sports participation. Only vigorous physical activity at leisure time was considered for this category.

A PA outcome was categorized as ‘active transport’ (AT) if the outcome measure was defined as walking or cycling to work, school or other destinations such as shops or friends.

For some studies, PA outcomes could not be clearly classified in either of these groups (e.g. heavy manual leisure (like chopping wood) or walking or cycling of which the purpose (leisure or transport) was not clear). Therefore, these outcomes were excluded from the current review.

#### Classification of the socioeconomic position indicators

The following guidelines were used to classify the SEP indicators in this study.

Income refers to (net or gross) individual income or household income. When area-level income was used as an indicator, it was classified as ‘other’ and specified further in the footnotes of the tables.

Education refers to the highest attained level of education (e.g. university education) or as the total years of education.

Social class refers to occupation-based social class, such as blue collar or white collar workers, or the British Registrar General classification [19].

Other SEP indicators that were included were neighbourhood SEP, such as mean/median income of a neighbourhood, material circumstances, such as home ownership, or other individual SEP measures, such as an individual composite SEP score that was constructed from several SEP indicators.

Parental SEP, childhood SEP, or the SEP of the spouse were excluded as a SEP indicator in this review.

#### Classification of European regions

The results were grouped by European region, based on geographical location and type of welfare regime [20,21]. The regions that were distinguished are:

Anglo-Saxon region, including Great-Britain and Ireland

Western European region, including Belgium, France, Germany, Luxembourg, Netherlands, and Switzerland

Scandinavian region, including Denmark, Finland, Norway, and Sweden

Southern European region, including Greece, Italy, Portugal, and Spain

Eastern European region, including Albania, Croatia (Hrvatska), Czech Republic, Estonia, Hungary, Latvia, Lithuania, Poland, and Slovenia

As many studies included more than one PA domain and/or more than one SEP indicator, the results were analysed on the level of the separate associations rather than the level of complete studies. This is in concordance with methods form McLaren [6] and Ball and Crawford [22]. The advantage is that we could distinguish between the domains of PA behaviour and the SEP indicators. Disadvantages of this method are that all associations are weighted equally and that studies with more associations have more influence than those with only one reported association [6].

Detailed tables in which all the associations reported in the included studies were synthesized are described in the additional tables ( Additional file 1, tables A1-A5, one for each domain of PA). A ‘+’ indicates a positive and significant association between the SEP indicator and the PA outcome of interest, a ‘-’ indicates a negative and significant association between the SEP indicator and the PA outcome of interest. A ‘0’ means that there was no significant (linear) association found. Significance was judged with α = 0.05. When there were more than two categories, the overall test of significance, or trend test was used (when available). If not available, significance was judged by looking at the significance level of the difference between the two most extreme groups. When there was no trend, or a curvilinear trend, for example when only the middle group was significantly different (but not the extremes), the association was classified as being non significant. When the symbol is between brackets, no test of significance was reported and difference was judged solely on descriptive measures such as percentages.

When both adjusted and unadjusted results were presented in the manuscripts, the adjusted results were recorded into the table, including a notification of the variables that were used for adjustment. Duplicate articles on the same study population were only included in the tables if they contributed unique associations not previously reported. Distributions of reported positive, negative, and null associations were evaluated by gender, SEP indicator, and European region for each PA outcome (Tables 2 and 3).

**Table 2:** **Distribution of positive, negative, and null associations by gender, SEP indicator, and PA domain**^**a**^


		**Total**				**Socioeconomic indicator**															
**Physical activity**^**b,c**^		**TOTAL**				**Income**				**Education**				**Social class**				**Other**			
			**+**	**0**	**-**		**+**	**0**	**-**		**+**	**0**	**-**		**+**	**0**	**-**		**+**	**0**	**-**
	**Gender**^**d**^	**n**	**%**	**%**	**%**	**n**	**%**	**%**	**%**	**n**	**%**	**%**	**%**	**n**	**%**	**%**	**%**	**n**	**%**	**%**	**%**
**TPA**	♂	**34**	**41%**	**24%**	**35%**	5	20%	60%	20%	16	50%	6%	44%	6	17%	17%	67%	7	57%	43%	0%
	♀	**36**	**39%**	**31%**	**31%**	5	0%	80%	20%	16	38%	25%	38%	6	17%	33%	50%	9	78%	11%	11%
	**all**	**70**	**40%**	**27%**	**33%**	**10**	**10%**	**70%**	**20%**	**32**	**44**%	**16**%	**41**%	**12**	**17**%	**25**%	**58**%	**16**	**69**%	**25**%	**6**%
**OPA**	♂	**10**	**10%**	**20%**	**70%**	1	0%	100%	0%	4	25%	25%	50%	4	0%	0%	100%	1	0%	0%	100%
	♀	**9**	**11%**	**33%**	**56%**	1	0%	100%	0%	4	25%	25%	50%	3	0%	33%	67%	1	0%	0%	100%
	**all**	**19**	**11%**	**26%**	**63%**	**2**	**0%**	**100%**	**0%**	**8**	**25**%	**25**%	**50**%	**7**	**0**%	**14**%	**86**%	**2**	**0**%	**0**%	**100**%
**TLTPA**	♂	**104**	**68%**	**31%**	**1%**	17	71%	29%	0%	56	68%	30%	2%	19	79%	21%	0%	12	50%	50%	0%
	♀	**96**	**68%**	**32%**	**0%**	17	47%	53%	0%	49	78%	22%	0%	19	68%	32%	0%	11	55%	45%	0%
	**all**	**200**	**68%**	**32%**	**1%**	**34**	**59%**	**41%**	**0%**	**105**	**72**%	**27**%	**1**%	**38**	**74**%	**26**%	**0**%	**23**	**52**%	**48**%	**0**%
**VLTPA**	♂	**56**	**75%**	**25%**	**0%**	12	83%	17%	0%	24	67%	33%	0%	10	80%	20%	0%	10	80%	20%	0%
	♀	**54**	**78%**	**22%**	**0%**	12	67%	33%	0%	24	75%	25%	0%	10	90%	10%	0%	8	88%	13%	0%
	**all**	**110**	**76%**	**24%**	**0%**	**24**	**75%**	**25%**	**0%**	**48**	**71**%	**29**%	**0**%	**20**	**85**%	**15**%	**0**%	**18**	**83**%	**17**%	**0**%
**AT**	♂	**26**	**35%**	**31%**	**35%**	4	25%	25%	50%	14	50%	36%	14%	3	33%	33%	33%	5	0%	20%	80%
	♀	**22**	**41%**	**27%**	**32%**	4	50%	0%	50%	12	58%	25%	17%	3	0%	67%	33%	3	0%	33%	67%
	**all**	**48**	**38%**	**29%**	**33%**	**8**	**38%**	**13%**	**50%**	**26**	**54**%	**31**%	**15**%	**6**	**17**%	**50**%	**33**%	**8**	**0**%	**25**%	**75**%

**Table 3:** **Distribution of positive, negative, and null associations by gender, European region, and PA domain**^**a**^


		**European region**																							
**Physical activity**^**b,c**^		**EU lackwide studies**				**Western European region**				**Eastern European region**				**Southern European region**				**Scandinavian region**				**Anglo-Saxon region**			
			**+**	**0**	**-**		**+**	**0**	**-**		**+**	**0**	**-**		**+**	**0**	**-**		**+**	**0**	**-**		**+**	**0**	**-**
	**Gender**^**d**^	**n**	**%**	**%**	**%**	**n**	**%**	**%**	**%**	**n**	**%**	**%**	**%**	**n**	**%**	**%**	**%**	**n**	**%**	**%**	**%**	**n**	**%**	**%**	**%**
**TPA**	♂	1	100%	0%	0%	1	0%	100%	0%	5	40%	40%	20%	6	17%	0%	83%	10	50%	10%	40%	11	45%	36%	18%
	♀	1	100%	0%	0%	1	0%	100%	0%	5	40%	20%	40%	6	17%	17%	67%	10	30%	40%	30%	13	54%	31%	15%
	**all**	**2**	**100%**	**0%**	**0%**	**2**	**0%**	**100%**	**0%**	**10**	**40**%	**30**%	**30**%	**12**	**17**%	**8**%	**75**%	**20**	**40**%	**25**%	**35**%	**24**	**50**%	**33**%	**17**%
**OPA**	♂	1	100%	0%	0%	-	-	-	-	3	0%	67%	33%	-	-	-	-	3	0%	0%	100%	3	0%	0%	100%
	♀	1	100%	0%	0%	-	-	-	-	3	0%	67%	33%	-	-	-	-	2	0%	0%	100%	3	0%	33%	67%
	**all**	**2**	**100%**	**0%**	**0%**	**-**	**-**	**-**	**-**	**6**	**0%**	**67**%	**33**%	**-**	**-**	**-**	**-**	**5**	**0%**	**0%**	**100%**	**6**	**0**%	**17**%	**83**%
**TLTPA**	♂	3	100%	0%	0%	20	75%	25%	0%	24	50%	46%	4%	14	71%	29%	0%	33	79%	21%	0%	10	50%	50%	0%
	♀	3	100%	0%	0%	17	88%	12%	0%	24	42%	58%	0%	14	71%	29%	0%	27	81%	19%	0%	11	45%	55%	0%
	**all**	**6**	**100%**	**0%**	**0%**	**37**	**81**%	**19**%	**0%**	**48**	**46**%	**52**%	**2**%	**28**	**71**%	**29**%	**0%**	**60**	**80**%	**20**%	**0**%	**21**	**48**%	**52**%	**0%**
**VLTPA**	♂	-	-	-	-	15	100%	0%	0%	11	64%	36%	0%	9	33%	67%	0%	8	88%	13%	0%	13	77%	23%	0%
	♀	-	-	-	-	13	92%	8%	0%	12	67%	33%	0%	10	40%	60%	0%	8	88%	13%	0%	11	100%	0%	0%
	**all**	**-**	**-**	**-**	**-**	**28**	**96**%	**4**%	**0%**	**23**	**65**%	**35**%	**0%**	**19**	**37**%	**63**%	**0%**	**16**	**88**%	**13**%	**0**%	**24**	**88**%	**13**%	**0%**
**AT**	♂	1	100%	0%	0%	7	29%	14%	57%	6	33%	17%	50%	-	-	-	-	6	50%	33%	17%	6	17%	67%	17%
	♀	1	100%	0%	0%	5	40%	20%	40%	6	33%	17%	50%	-	-	-	-	6	50%	33%	17%	4	25%	50%	25%
	**all**	**2**	**100%**	**0%**	**0%**	**12**	**33**%	**17**%	**50**%	**12**	**33**%	**17**%	**50**%	**-**	**-**	**-**	**-**	**12**	**50**%	**33**%	**17**%	**10**	**20**%	**60**%	**20**%

#### Quality assessment

Since only observational studies were included in this study, methods for quality assessment were limited. Only a few basic quality guidelines were used as exclusion criteria. All included studies were treated equally in the results. To check if quality issues affected the results, sensitivity analyses were conducted for three common quality markers; response, adjustment, and sample size. In these analyses, the results were synthesized again after excluding the articles that did not report a response or studies with a response of less than 50%. In separate analysis, associations that were not adjusted for at least age and gender were excluded from the results. Finally, the results were synthesized for those studies with at least 2000 participants. The results that were found in the subsets of associations were compared with the results obtained when all publications were included.

## Results

**Figure 1:**
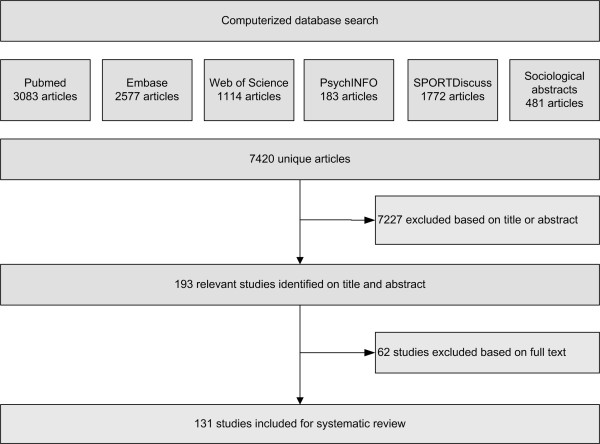
Flowchart of search and selection process.

The search strategy retrieved 7420 unique and potentially relevant titles (Figure 1). After scanning titles and abstracts a total of 193 articles were identified for inclusion. Sixty-two articles were excluded, primarily because no association between SEP and PA was reported (n = 18), because of quality concerns (n = 11), because the population was older than 65 (n = 8), or because the study was conducted outside of Europe (n = 6). As a result, 131 studies were included in the current review.

These 131 studies reported on 105 study populations and 447 unique associations between a SEP indicator and PA outcome (Table 1). Most studies were conducted in Scandinavian countries and Great Britain. The majority of the sample sizes were large (e.g. including over 4000 participants) with a range from 224 to 60 938 participants. In most studies the response was higher than 60% (range 20-96%) but approximately one quarter of the studies did not report any response percentage. Apart from the study by Van Dyck and colleagues [23] who used accelerometer data in addition to self-reported data, all studies relied on self-reported PA. The majority of the studies did not report the validity of the PA measure. The most frequently used validated PA questionnaire was the International PA Questionnaire (IPAQ) [24], other validated measures that were used were the Minnesota Leisure Time PA Questionnaire [25], the MONICA Optional Study of PA Questionnaire (MOSPA-Q) [26], the Short Questionnaire to Assess Health-Enhancing PA (SQUASH) [27], and the Modifiable Activity Questionnaire (MAQ) [26].

### Total physical activity

There were 30 studies, with a total of 70 unique associations, which reported on the association between SEP IMDStril’ total PA ( Additional file 1, Table A1). Approximately equal amounts of positive (n = 28), null (n = 19) associations, and negative (n = 23) associations were found (Table). This pattern did not differ between men and women. While most associations were not statistically significant with income as indicator of SEP, both positive and negative associations were found with education as indicator of SEP (Table 2). In Southern Europe, nine out of 12 assessed associations (75%) indicated decreasing levels of physical activity by increasing levels of SEP, while in the Anglo-Saxon countries most (50%) associations showed the opposite pattern (Table 3).

### Occupational physical activity

There were 10 studies, with a total of 19 unique associations, which reported on the association between SEP and occupational PA ( Additional file 1, Table A2). The majority of the associations (68%) were negative, indicating that persons in lower socioeconomic groups did more occupational PA (Table 2). Patterns were similar for men and women. Almost all associations based on social class showed a negative relationship, while mixed patterns were found for education and income (Table 2). In studies in Eastern Europe, four out of six associations were non significant, while mainly negative associations were found in other regions of Europe (Table 3).

### Leisure-time physical activity

Leisure-time PA was the most frequent domain of PA assessed in relation to SEP. A total of 112 studies reported 310 unique associations. The results are presented for total leisure-time PA and vigorous leisure-time PA separately.

#### Total leisure-time physical activity

A total of 75 studies reported 200 unique associations ( Additional file 1, Table A3) on the association between TLTPA and SEP. Most studies (68% of associations) showed that people with a higher SEP were more likely to be physically active in their leisure-time, whereas one study reported that a higher SEP was associated with less TLTPA (Table 2). The association between education and TLTPA was reported most frequently and most studies found a positive association (74%) (Table 2). Men and women differed slightly by the SEP indicator used. For women, the associations between education and TLTPA were mostly positive (78% in women versus 68% in men), and for men the associations between social class and TLTPA were mostly positive (79% in men versus 68% in women). Income showed a more consistent positive association with TLTPA among men (71% positive) compared to women (47% positive). There were also geographical differences (Table 3). In Scandinavia and the Western European countries, predominantly positive associations were observed (84% and 81% respectively). In Eastern Europe and in the Anglo-Saxon region, only half of the associations were positive (46% and 48% respectively), with the remaining being null associations.

#### Vigorous leisure-time physical activity

The results from the 37 included studies reporting about VLTPA and SEP showed clear socioeconomic inequalities in VLTPA ( Additional file 1, Table A4). A total of 84 out of the 110 associations (76%) were positive, indicating that higher socioeconomic groups were more vigorously physically active during leisure-time than lower socioeconomic groups (Table 2). No studies found a significant inverse association. Income was found to be positively associated with VLTPA more frequently among men (83%) than among women (67%) (Table 2). Regarding the other SEP indicators, the results were slightly more pronounced in women. Nearly all studies (96%) conducted in the Western European region reported that VLTPA was more prevalent among people with a higher SEP (Table 3). In both Scandinavia and in the Anglo-Saxon countries, the positive associations also dominated (both 88% positive), whereas in Southern Europe about a third of the associations were positive (37%), the other 63% being non significant.

### Active transport

There were 11 studies that examined socioeconomic differences in active transport ( Additional file 1, Table A5). Two studies distinguished between engaging in active transport (yes/no) and the amount of active transport in a week [28,29]. This resulted in a total of 48 associations of which 18 (38%) were positive, 14 (29%) were neutral, and 16 (33%) were negative (Table 2). There were no clear differences by gender, SEP indicator, or geographic region (Tables 2 and 3).

### Quality sensitivity analyses

After excluding all studies that did not report a percentage of response or that did not have a response of at least 50% (n = 40), a total of 91 studies remained in the sensitivity analysis. The number of associations decreased from 447 to 313, though patterns remained similar ( Additional file 1, Table A6 and Table A7). The main difference was that now all associations between OPA and SEP were negative, compared with 63% in the main analysis.

Excluding associations that were not at least adjusted for age and gender from the analysis resulted in a total of 342 unique associations ( Additional file 1, Table A6 and Table A7). In this restricted set of studies, all associations between OPA and SEP were negative thus accentuating the negative pattern found in the main analysis. All other patterns remained similar.

Finally, excluding the studies with less than 2000 participants (n = 31) resulted in an analysis with the remaining 100 studies ( Additional file 1, Table A6 and Table A7). The patterns became somewhat more pronounced, since larger studies in general produce more significant associations. In this restricted set of studies, half of the associations for TPA were positive, compared with 40% in the main analysis. Also the associations in TLTPA and VLTPA were more often positive (77% and 82% relatively compared with 68% and 76%). The associations between OPA and SEP were more often negative (77% compared with 63%). The pattern for active transport remained similar.

## Discussion

Patterns of socioeconomic inequalities in PA are perhaps more complex than often thought. The direction of socioeconomic inequalities in PA in Europe differs considerably by domain of PA and to some degree by European region and socioeconomic indicator. Since only few studies reported men and women separately, no conclusions about gender differences are warranted.

### Domains of physical activity

Different domains of PA demonstrated different socioeconomic patterns. The most consistent socioeconomic inequalities were found for vigorous leisure-time PA, with the lower SEP groups participating less in vigorous activities like sports than higher SEP groups. For overall leisure-time PA a similar pattern was observed although less articulated. In contrast to PA during leisure time, occupational PA was more frequently reported by lower SEP groups. For total PA and active transport, many studies found a significant association, but they differed considerably in direction.

The absence of a consistent direction in the socioeconomic inequalities in total PA might be caused by the contrasting socioeconomic patterns found for leisure-time PA and occupational PA, that both may make up a large part of total PA. This was nicely illustrated by a study by Lissner and colleagues [30]. They studied leisure-time PA, occupational PA, and PA index (total PA) which was a combined measure of occupational and leisure-time PA. Their results showed that education was positively associated with leisure-time PA and inversely associated with occupational PA. Education and the PA index were not associated since the association between leisure-time PA and occupational PA evened each other out. This mechanism may partly explain the contradictory results with as much negative as positive associations between SEP and total PA, since the association will be determined by the relative influence of leisure-time PA and occupational PA on total PA.

Another question that rises is whether occupational physical activity compensates for not being active during leisure time. A few included studies [31,32] examined socio-economic differences in leisure-time PA while correcting for occupational PA. In the multivariable models, both income and education, and occupational PA were significantly associated with leisure-time PA. These studies indicated that although respondents who were more occupationally active were less active in leisure time, people from lower socio-economic backgrounds were still less physically active compared to high socio-economic people, even after correcting for occupational PA.

Also, by including occupational PA as an indicator of healthy PA, it is assumed that occupational PA is beneficial to health, however this may not be the case [33]. The few studies that look at associations between occupational PA and mortality or morbidity show no clear pattern. There are studies that report a beneficial effect [34-38], no effect [39], or a detrimental effect [40-43] of occupational PA on cardiovascular diseases and mortality. The health benefits of leisure-time PA and sports are more consistent [34-37,42,44,45]. The different types of activity carried out at work might partly explain these inconsistent findings. For example, Fransson *et al.*[46] found that walking and standing at work, both aerobic activities, decreases the risk of myocardial infarction, while lifting or carrying at work increases the risk of myocardial infarction. The relation between all aspects of occupational PA and health should be investigated further.

Active transport was studied considerably less often than the other domains of PA and no clear pattern was detected. There were almost equal amounts of studies showing a positive, a null, or a negative association between SEP and active transport. It could be that whether or not one engages in active transport and time spent doing so have different determinants. The two studies that distinguished between participation and time spent in active transport showed for example that participation was not or inversely associated with education while, among the participators, the higher educated spent more time in active transport [28,29]. The contradictory results may also be explained by factors that influence the association between SEP and active transport. A Dutch and a Belgium study both looked at neighbourhood SEP as an indicator of active transport and found negative associations [23,47]. This could either be an indication that people with a lower SEP are more likely to engage in transport PA or for example, that neighbourhoods with a low SEP are more likely to make people engage in transport PA for example because of a higher density or more connectivity [48]. External factors such as connectivity, density and the availability of public transport might be especially important for active transport PA and more research should be conducted to get a better insight into determinants of active transport.

### Types of SEP indicator

Income, education and occupation reflect different aspects of SEP [17,18]. Occupational class appears to be the SEP indicator most sensitive for studying SEP differences in occupational PA. However, the consistent associations found for this indicator may also be due to the definitions used to describe social classes. Because manual jobs are in general considered to be of lower social class, the social class definition is often partly based on having a manual or a non-manual job. This already implies a difference in activities at work.

Inequalities in leisure-time PA and vigorous activity are often thought to be caused by either an educational effect on knowledge about the positive health consequences of PA, or financial possibilities to engage in leisure-time PA, for example to buy PA equipment or to afford memberships or admission rates for sports and PA facilities. The fact that the patterns in inequalities in PA were roughly similar for the different indicators of SEP, including education and income, suggest that it is not one or the other but both may indeed be important. Other factors related to chance and choice of lifestyle [49], such as SEP differences in social or cultural capital [50] or differences in physical environmental opportunities for PA [51,52], may be additional determinants of SEP inequalities in PA. Also, some factors, such as intrapersonal factors, may act as intermediary in the process between SES and PA [52]. In a previous review, Gidlow and colleagues [53] reported that education was stronger associated with PA than income. Although in the present review education was the most frequent studied SEP indicator we could not confirm that the associations of education with PA were also stronger than the associations with the other SEP indicators.

### European regions

A recent study showed that the largest inequalities in obesity prevalence were found in Southern Europe, especially among women, and the smallest in Eastern Europe [7]. In concordance with these findings, we found that the socioeconomic inequalities in PA were less consistent in Eastern Europe for both occupational PA and leisure-time PA. Opposite to what would be expected from the inequalities found in obesity, the inequalities in vigorous leisure-time PA were least pronounced in Southern Europe. This was also found in the few pan-European studies that were included in this review [54-56] and by a recent pan-European study by Mäkinen *et al.*[57]. A possible explanation could be that general levels of PA are low in these countries [54,57] which would make it harder to detect SEP differences in PA.

### Strengths & limitations

The main strength of this review is the systematic exploration of different domains of physical activity, different SEP indicators, and geographic regions of Europe. Also, the inclusion of a quality sensitivity analyses strengthens the results. There are, however, also some limitations to be taken into account when interpreting the results.

Like any review of the published literature, the present review may suffer from publication bias [58]. The fact that a substantial numbers of null findings were reported in the reviewed studies may indicate that publication bias may not be severe. Also, some relevant studies may have been missed because only English-language studies that were available in electronic databases and that were published in peer-reviewed journals were included. Moreover, by analyzing the data on the level of the associations instead of the level of studies, more weight was given to studies that reported more than one association. Although this may have influenced conclusions based on all reported associations, this influence was expected to be smaller when subgroups of associations, such as by PA domain and SEP indicator, are considered.

Methodological differences between the included studies, such as the assessment of PA [59], the selection of participants, and the adjustment for confounders, could have influenced the reported associations. Although this probably introduced some noise, the sensitivity analysis showed that the overall patterns seem to be quite stable.

## Conclusion

This review showed that leisure-time PA, and specifically vigorous leisure-time PA, is less prevalent while occupational PA is more prevalent among people with lower SEP. Although there were some regional differences, these inequalities were visible throughout Europe. The contradictory inequalities for total PA may partly be explained by the contrasting socioeconomic patterns found for leisure-time PA and occupational PA. These inconsistent results in total PA indicate that total PA may not be a suitable summary measure when investigating inequalities in PA and their effects on morbidity and mortality.

The found inequalities indicate that leisure-time PA should be an important focus in improving physical activity levels and reducing inequalities. However, interventions aimed at improving leisure-time PA in lower socioeconomic groups needs to acknowledge their potential higher levels of occupational PA.

## Abbreviations

PA: Physical Activity; MET: Metabolic Equivalent; SEP: Socioeconomic Position; TPA: Total physical Activity; OPA: Occupational Physical Activity; TLTPA: Total Leisure Time Physical Activity; VLTPA: Vigorous Leisure Time Physical Activity; AT: Active Transport.

## Competing interest

The author(s) declare that they have no competing interests'.

## Authors’ contributions

MAB carried out the systematic literature search, the title scanning and selection, the data synthesis, and the drafting of the manuscript. CBMK assisted in the title selection and the data synthesis. She also helped to interpret the results and critically reviewed and improved the manuscript. KG, FJvL, and AEK designed the study, helped to interpret the results, and critically reviewed and improved the manuscript. JB and AB helped to interpret the results and critically reviewed and improved the manuscript. All authors read and approved the final manuscript.

## Supplementary Material

Additional file 1 **Table A1.** Summary of study findings examining associations between total or usual physical activity (TPA) and SEP. Table A2: Summary of study findings examining associations between occupational physical activity (OPA) and SEP. Table A3: Summary of study findings examining associations between total leisure-time physical activity (TLTPA) and SEP. Table A4: Summary of study findings examining associations between vigorous leisure-time physical activity (VLTPA) and SEP. Table A5: Summary of study findings examining associations between active transport (AT) and SEP. Table A6: Distribution of positive, negative, and null associations by SEP indicator and PA domain in differenet subsets of the reviewed associations. Table A7: Distribution of positive, negative, and null associations by European region and PA domain in differenet subsets of the reviewed associations. Click here for file
