# Supplementary material for: Socioeconomic inequalities in occupational, leisure-time, and transport related physical activity among European adults: A systematic review
Source: Int J Behav Nutr Phys Act. 2012 Sep 19;9:116. doi: 10.1186/1479-5868-9-116 (PMC3491027; doi:10.1186/1479-5868-9-116)
Supplement: Additional file 1 — Table A1. Summary of study findings examining associations between total or usual physical activity (TPA) and SEP. Table A2: Summary of study findings examining associations between occupational physical activity (OPA) and SEP. Table A3: Summary of study findings examining associations between total leisure-time physical activity (TLTPA) and SEP. Table A4: Summary of study findings examining associations between vigorous leisure-time physical activity (VLTPA) and SEP. Table A5: Summary of study findings examining associations between active transport (AT) and SEP. Table A6: Distribution of positive, negative, and null associations by SEP indicator and PA domain in differenet subsets of the reviewed associations. Table A7: Distribution of positive, negative, and null associations by European region and PA domain in differenet subsets of the reviewed associations. [file 1479-5868-9-116-S1.pdf]

Title:

Socioeconomic inequalities in occupational, leisure-time, and transport related physical activity among European adults: A systematic review.

Journal:

International Journal of Behavioral Nutrition and Physical Activity

Authors:

Beenackers, M.A. [a] *

Kamphuis, C.B.M. [a]

Giskes, K. [a,b,c]

Brug, J. [d]

Kunst, A.E. [a, e]

Burdorf, A. [a]

van Lenthe, F.J. [a]

Affiliations of authors:

a Department of Public Health, Erasmus MC, Rotterdam, Netherlands.

b School of Public Health/Institute for Health and Biomedical Innovation, Queensland University of Technology, Brisbane, Australia.

c School of Medicine, University of Sydney, Sydney, Australia.

d Department of Epidemiology & Biostatistics and the EMGO Institute for Health and Care Research, VU University Medical Centre, Amsterdam, Netherlands

e Department of Public Health, AMC, University of Amsterdam

*** Corresponding author:**

E-mail: m.beenackers@erasmusmc.nl

Content of Additional file 1

Table A1: Summary of study findings examining associations between total or usual physical activity (TPA) and SEP 3

Table A2: Summary of study findings examining associations between occupational physical activity (OPA) and SEP 5

Table A3: Summary of study findings examining associations between total leisure-time physical activity (TLTPA) and SEP 6

Table A4: Summary of study findings examining associations between vigorous leisure-time physical activity (VLTPA) and SEP 10

Table A5: Summary of study findings examining associations between active transport (AT) and SEP 12

Table A6: Distribution of positive, negative, and null associations by SEP indicator and PA domain in different subsets of the reviewed associations 13

Table A7: Distribution of positive, negative, and null associations by European region and PA domain in different subsets of the reviewed associations 14

Note: a list of the references can be found in the article

Table A1: Summary of study findings examining associations between total or usual physical activity (TPA) and SEP[‡]

**Author (Date)**	**Direction of the associations between the socioeconomic indicator and physical activity**								
	**Men**				**Women**				**Adjustment**
	**Income**	**Education**	**Social class**	**Other**	**Income**	**Education**	**Social class**	**Other**	
**EU wide studies**									
Ståhl *et al*, 2001[60]		+				+			age, gender, country, several cognitive factors
**Western European region**									
de Vries *et al*, 2008[78]		(0)				(0)			-
**Eastern European region**									
Frömel *et al*, 2009[80]				0[1]				-[1]	age, gender[s], BMI, smoking, # residents in town, living status, dog ownership, participating in organized PA
Jurakić *et al*, 2009[81]	0	-			0	-			-
Zaletel-Kragelj *et al*, 2006[91]		+	+			+	+		age, gender, education, kind of work, social class, residence community, geographical region
**Southern European region**									
Borrel *et al*, 2000b[95] [a]			-				0		age, gender[s]
Gal *et al*, 2005[97]		-	-			-	-		age, gender[s]
Panagiotakos *et al*, 2008[101] [b]		+				+			-
Santos *et al*, 2009[105]	-	-			-	-			gender[s]
**Scandinavian region**									
Barengo *et al*, 2006[109] [†]		-				0			age, gender[s]
Bergman *et al*, 2008[110]	0	-			0	-			age, gender, education, income, employment status, BMI, residential community size, marital status, smoking, self-perceived health
Kivimäki *et al*, 2007[116]				(+)[1]				(+)[1]	-
Lagerros *et al*, 2009[120]		-				-			-
Leijon *et al*, 2010[121]		+		+[3]		+		+[3]	survey design, non-response
Norman *et al*, 2002[131]		-							age, gender[s], BMI, smoking, marital status, self-rated health
Novak *et al*, 2006[132]		+				0			age[t], gender[s]
Orsini *et al*, 2007[133]						-			age, gender[s], BMI, postmenopausal status, alcohol consumption, smoking, employment status, urban/rural place in childhood
Pudaric *et al*, 2000[139]	+				0				gender[s]
**Anglo-Saxon region**									
Adams, 2009[151]		+				+			age, gender, self-reported health
Allender *et al*, 2008[15]		+	-			0	-		age, gender[s], education, social status, employment status
Amuzu *et al*, 2009[152] [c]								+[1], +[2]	gender[s], individual SEP, neighb. SEP
Chaudhury & Shelton, 2010[155] [d]	0		0	0	0		0	0[2]	age, gender[s], income, social class, employment status, long-term illness, BMI, waist circumference, waist-hip ratio, smoking status, alcohol consumption, region, deprivation index
Harrison *et al*, 2006[157]				+[4], +[2]				+[4], +[2]	age, gender
Mullineaux *et al*, 2001[164]		+				+			age, self perception of being active, motivation to be active, self perception of doing adequate exercise, lifestyle problems

Author (Date)	Direction of the associations between the socioeconomic indicator and physical activity								
	Men				Women				Adjustment
	Income	Education	Social class	Other	Income	Education	Social class	Other	
Myint *et al*, 2006[166] [e]			-				-		age, gender[s]
Popham & Mitchell, 2007[16]				0				+[1]	age, gender[s], health status, employment status

‡ The symbols in the table should be read as follows: '+' means that a high SEP is associated with a high TPA, a '-' means that a high SEP is associated with a low TPA. A '0' means that there was no association found. When the symbol is between brackets, no tests of significance were done.

† Results from most recent year (1997) reported. Associations in previous years were different.

a. Similar results found in the same sample population by Borrel *et al*, 2000a[94].

b. Similar results found in the same sample population by Panagiotakos *et al*, 2008a[100].

c. Similar results found in the same sample population by Hillsdon *et al*, 2008[159] and by Watt *et al*, 2009[173].

d. The association between social class and TPA was also reported by Allender *et al*, 2008[15] but since Chaudhury and Shelton[155] report about an older subsample (age 60-69), the results were different and therefore reported here as well.

e. Only the results for people under 65 are presented here.

s. Results were stratified according to gender.

t. All respondents were the same age.

1. Individual SEP (composite measure).
2. Neighbourhood SEP.
3. Self-reported economy.
4. Home ownership.

Table A1: Summary of study findings examining associations between <u>occupational physical activity (OPA)</u> and SEP‡

Author (Date)	Direction of the associations between the socioeconomic indicator and physical activity								
	Men				Women				Adjustment
	Income	Education	Social class	Other	Income	Education	Social class	Other	
**EU wide studies**									
Van Tuyckom & Scheerder, 2010b[55]		+				+			-
**Eastern European region**									
Jurakić *et al*, 2009[81]	0	0			0	0			-
Mišigoj-Durakoviæ *et al*, 2000[84]		-				-			gender[s]
**Scandinavian region**									
Mäkinen *et al*, 2010a[126]			-				-		age, gender[s]
Suadicani *et al*, 2001[42]			-						age, gender[s]
Wang *et al*, 2010[34] [a]		-				-			age, gender[s], study year
**Anglo-Saxon region**									
Mutrie & Hannah, 2004[165]			-				0		gender[s]
Poortinga, 2007[167]			-				-		-
Popham & Mitchell, 2007[16]				-[1]				-[1]	age, gender[s], health status, employment status

‡ The symbols in the table should be read as follows: '+' means that a high SEP is associated with a high OPA, a '-' means that a high SEP is associated with a low OPA. A '0' means that there was no association found. When the symbol is between brackets, no tests of significance were done.

a. Similar results found in the same sample population by Hu *et al*, 2003[37].

s. Results were stratified according to gender.

1. Composite individual SEP measure.

Table A3: Summary of study findings examining associations between <u>total leisure-time physical activity (TLTPA)</u> and SEP[‡]

Author (Date)	Direction of the associations between the socioeconomic indicator and physical activity								
	Men				Women				Adjustment
	Income	Education	Social class	Other	Income	Education	Social class	Other	
**EU wide studies**									
Van Tuyckom & Scheerder, 2010a[62] [a]			+				+		age, gender, marital status, geographical status
Van Tuyckom & Scheerder, 2010b[55] [a]		+				+			-
Varo *et al*, 2003[56] [b]		+				+			age, gender[s], education, marital status, smoking, weight change
**Western European region**									
Bertrais *et al*, 2004[64]		0				+			age, gender[s], smoking, urban/rural location, TV viewing
Chaix & Chauvin, 2003[65]	+	+			+	+			age, gender, education, income, occupation, employment, marital status
Dragano *et al*, 2007[66] - DEU		+		+[5]		+		+[5]	age, gender, education, economic activity, social isolation, neighbourhood unemployment
Drieskens *et al*, 2010[67]		(+)				(+)			gender[s]
Kamphuis *et al*, 2009[51]	0	+			0	+			age, gender
van Lenthe *et al*, 2005[47]				+ [2]				+ [2]	age, gender, educational level
van Oort *et al*, 2004[71]		(+)				(+)			age, gender
Rathmann *et al*, 2005[72]	+	0	+		+	+	+		age, gender[s]
Ribet *et al*, 2001[73] – GAZEL			+				+		age, gender[s], own and spouses occupation
Ribet *et al*, 2001[73] – MONICA			+				+		age, gender[s], own and spouses occupation
Van Dyck *et al*, 2010[23]		+		0 [2]		+		0 [2]	age, gender, education, employment status, BMI, walkability of neighbourhood, neighbourhood SEP, walkability*neighbourhood SEP
Verdaet *et al*, 2004[77]		+							gender[s]
Wagner *et al*, 2003[79] - FRA		0		+ [3]					age, gender[s], marital status, obesity, reported disease, centre
**Eastern European region**									
Dragano *et al*, 2007[66] – CZE		+		0[5]		+		0[5]	age, gender, education, economic activity, social isolation, neighbourhood unemployment
Drygas *et al*, 2009[31]	0	+			+	+			age, gender[s], place of residence, education, income, marital status, active commuting, occupational PA
Jurakić *et al*, 2009[81]	+	0			+	0			-
Kaleta & Jegier, 2005[32] [m]	+	+			0	+			age, gender[s], education, income, job characteristics (mental/physical work), occupational PA
Kaleta & Jegier, 2007[82] [m]	0	+			+	+			age, gender[s], education, income, job characteristics (mental/physical work), occupational PA
Mišigoj-Durakoviæ *et al*, 2000[84]<45 years		0				0			gender[s]
Mišigoj-Durakoviæ *et al*, 2000[84] >45 years		-				0			gender[s]
Pomerleau *et al*, 2000[87] – EST	+	+			0	+			age, gender[s], education, income, nationality, area of residence
Pomerleau *et al*, 2000[87] – LTU	+	0			0	+			age, gender[s], education, income, nationality, area of residence
Pomerleau *et al*, 2000[87] – LVA	+	+			0	+			age, gender[s], education, income, nationality, area of residence

Author (Date)	Direction of the associations between the socioeconomic indicator and physical activity								
	Men				Women				Adjustment
	Income	Education	Social class	Other	Income	Education	Social class	Other	
Puska *et al*, 2003[88] – EST		0				0			age, gender^{s}, place of residence, year of measurement
Puska *et al*, 2003[88] – LTU		0				0			age, gender^{s}, place of residence, year of measurement
Shapo *et al*, 2004[89]	0	0			0	0			age, gender^{s}, education, income, smoking, vegetable intake, alcohol intake, # unhealthy behaviours
Stelmach *et al*, 2004[90]	+	0			0	0			age, gender^{s}, education, income, marital status, chronic illnesses, self-rated health
**Southern European region**									
Bolívar *et al*, 2010[93]				0^{6}				0^{6}	age, gender^{s}, unemployment in municipality, illiteracy in municipality
Bolívar *et al*, 2010[93]		+	0	0^{5},$+^{7}$		+	+	0^{5},$+^{7}$	age, gender^{s}, education, occupational class, employment, children <15yrs, status, obesity, smoking status, self-rated health, sufficient green spaces in neighbourhood, unemployment in municipality, illiteracy in municipality
De Vogli *et al*, 2005[96]			+				+		gender^{s}
Gal *et al*, 2005[97]		+	+			+	+		age, gender^{s}
Meseguer *et al*, 2009[99]		+				+			age, gender, occupational physical activity
Pascual *et al*, 2007[102]	+	+	+	$+^{2}$	0	+	+	$+^{2}$	age, gender^{s}, education, social class, income, neighbourhood income, # sports facilities/1000 inhabitants, rurality, density
Schröder *et al*, 2004[106]		0				0			gender^{s}
**Scandinavian region**									
Henriksson *et al*, 2003[115]		+							gender^{s}
Korniloff *et al*, 2010[117]	+	+			+	+			age, gender, education, income, marital status, smoking, presence of metabolic syndrome or depressive symptoms
Lahelma *et al*, 2010[160] – FIN			+				0		age, gender^{s}, job strain, working overtime, work-family conflicts, marital status, social relations
Lindström *et al*, 2001[122]			+				+		age, gender^{s}, country of origin, previous/current diseases, social participation
Lindström *et al*, 2003a[123] ^{c}		+				+			-
Mäkinen *et al*, 2009[125] $^{d, e}$	+	+	0		+	+	0		Age, gender^{s}, income, education, occupation
Mäkinen *et al*, 2010b[127] ^{f}	+	+	+		+	+	+		age, gender^{s}
Molarius, 2003[129]		(+)				(+)			gender^{s}
Nielsen *et al*, 2006[130]		+							age, gender^{s}
Norman *et al*, 2002[131]		0							age, gender^{s}, BMI, smoking, marital status, self-rated health
Osler *et al*, 2001[135]		0				0			age, gender^{s}, own and parent's education, occupational physical activity, smoking status
Osler *et al*, 2000[134]		0				+			age, gender^{s}, height, weight, BMI, SPB, DPB, Total cholesterol, HDL cholesterol, smoking, heavy smoking, healthy eating, multiple risk index
Osler *et al*, 2008[136]		+							age^{t}, gender^{s}, father's social class, cognitive function, labour market participation, divorce
Petersen *et al*, 2010[137]		+				+			age, gender^{s}

Author (Date)	Direction of the associations between the socioeconomic indicator and physical activity								
	Men				Women				Adjustment
	Income	Education	Social class	Other	Income	Education	Social class	Other	
Pulkki *et al*, 2003a[140]				0[1]				+[1]	age, gender[s],
Pulkki *et al*, 2003b[141]		0				+			age, gender[s], parental education
Puska *et al*, 2003[88] – FIN		+				0			age, gender[s], place of residence, year of measurement
Salonen *et al*, 2010[142]		+	+				0		age, gender[s]
Schnohr *et al*, 2004[143] [g]		+				+			gender[s]
Sjögren & Stjernberg, 2010[145]		0				+			age, gender[s], being able to bath or shower independently, age*having access to areas for country walks
Strand & Tverdal, 2004[146]		+				+			gender[s]
Strandhagen *et al*, 2010[147]		+				+			age, gender[s]
Suadicani *et al*, 2005[148] [h]			+						gender[s]
Tammelin *et al*, 2003[149] [i]		+				+			age[t], gender[s], children, education, employment status, place of residence, several sports related factors at age 14
Wang *et al*, 2010[34] [j]		+				+			age, gender[s], study year
Wemme & Rosvall, 2005[150] [k]		+	+			+	+		age, gender[s]
**Anglo-Saxon region**									
Allender *et al*, 2008[15]		+	0			+	0		age, gender[s], education, work activity status, social status
Heslop *et al*, 2001[158]						0	0	0[2]	age, gender[s]
Mein *et al*, 2005[163] [l]			+				+		age, gender[s], marital status, month of questionnaire completion
Mutrie & Hannah, 2004[165]			+				+		gender[s]
Popham & Mitchell, 2006[168]	0	0	0	0[4]	0	+	0	+[4]	age, gender[s], income, education, occupation, school type, marital status, self-rated health, general health questionnaire, health limits daily activities, GP consultations, smoking, year, housework hours, employment status, children
Wagner *et al*, 2003[79] - IRE		+		+[3]					age, gender[s], marital status, obesity, reported disease

‡ The symbols in the table should be read as follows: '+' means that a high SEP is associated with a high TLTPA, a '-' means that a high SEP is associated with a low TLTPA. A '0' means that there was no association found. When the symbol is between brackets, no tests of significance were done.

a. Similar results found in the same sample population by Van Tuyckom & Scheerder, 2008[61].
b. Similar results found in the same sample population by Martinez-Gonzalez *et al*, 2001[54].
c. Similar results found in the same sample population by Lindström *et al*, 2003b[124].
d. Results from the most recent data were reported (1998-2002).
e. Similar results found in the same sample population by Laaksonen *et al*, 2002[118] and Laaksonen *et al*, 2008[119].
f. Similar results found in the same sample population by Mäkinen *et al*, 2010a[126].
g. Similar results found in the same sample population by Andersen *et al*, 2000[108].
h. Similar results found in the same sample population by Suadicani *et al*, 2001[42].
i. Similar results found in the same sample population by Häkinnen *et al*, 2006[114].
j. Similar results found in the same sample population by Borodulin *et al*, 2008[111], Mäkinen *et al*, 2010c[128], and Hu *et al*, 2003[37].
k. Similar results found in the same sample population by Ali & Lindström, 2006[107].

l. Similar results found in the same sample population by Stringhini *et al*, 2010[3], Bartley *et al*, 2004[154], and Lahelma *et al*, 2010[160] (data from Great-Britain).
m. Both studies of Kaleta & Jegier[32, 82] are reported here. Although they report the same association in a similar dataset, they provide different results with respect to the association between income and TLTPA in the adjusted model.
s. Results were stratified according to gender.
t. All respondents were the same age.
1. Composite individual SEP measure.
2. Neighbourhood SEP.
3. Household wealth (material conditions).
4. Fee paying versus public school.
5. % unemployment in neighbourhood/municipality.
6. Economic level of municipality.
7. Illiteracy in municipality.

Table A4: Summary of study findings examining associations between <u>vigorous leisure-time physical activity (VLTPA)</u> and SEP[‡]

**Author (Date)**	**Direction of the associations between the socioeconomic indicator and physical activity**								
	**Men**				**Women**				**Adjustment**
	**Income**	**Education**	**Social class**	**Other**	**Income**	**Education**	**Social class**	**Other**	
**Western European region**									
Addor *et al*, 2003[63] – <45 years		**+**				**0**			gender[s]
Addor *et al*, 2003[63] – 45+ years		**+**				**+**			gender[s]
Galobardes *et al*, 2003[68]			**+**				**+**		age, gender[s]
Kamphuis *et al*, 2008[52]	**+**	**+**			**+**	**+**			age, gender
van Lenthe *et al*, 2005[47]				**+**[2]				**+**[2]	age, gender, educational level
Meyer *et al*, 2005[69]	**+**	**+**			**+**	**+**			age, gender, education, income, size of household, urban/rural residence, language
Nocon *et al*, 2008[70] [a]	**+**	**+**	**+**		**+**	**+**	**+**		age, gender, income, education, occupation
Scheerder *et al*, 2005[75] [b]		**+**	**+**			**+**	**+**		gender[s]
Wagner *et al*, 2003[79] - FRA		**+**		**+**[3]					age, gender[s], marital status, obesity, reported disease, centre
**Eastern European region**									
Frömel *et al*, 2009[80]				**0**[1]				**0**	age, gender[s], BMI, smoking, # residents in town, living status, dog ownership, participating in organized PA
Leskosek *et al*, 2002[83]		**+**				**+**			age, gender, employment, marital status/family status
Mišigoj-Durakoviæ *et al*, 2000[84]		**+**				**+**			age[†], gender[s],
Nowak, 2010[85]						**+**			gender[s]
Paulik *et al*, 2010[86]		**+**		**+** [3]		**+**		**+** [3]	-
Pomerleau *et al*, 2000[87] – EST	**+**	**0**			**+**	**+**			age, gender[s], nationality (native, Russian, other), area of residence (rural, urban), education, income
Pomerleau *et al*, 2000[87] – LTU	**+**	**0**			**0**	**+**			age, gender[s], nationality (native, Russian, other), area of residence (rural, urban), education, income
Pomerleau *et al*, 2000[87] – LVA	**+**	**0**			**0**	**0**			age, gender[s], nationality (native, Russian, other), area of residence (rural, urban), education, income
**Southern European region**									
Artazcoz *et al*, 2004[92]						**(+)**			gender[s]
Borrel *et al*, 2000a[94]			**0**				**0**		age, gender[s], employment status, family structure
Lera-López & Rapún-Gárate, 2005[98]	**0**	**0**			**0**	**0**			age, gender, education, size of household, income, degree of urbanization, employment status
Pascual *et al*, 2009[103]	**+**	**+**			**+**	**+**			age, gender[s]
Pitsavos *et al*, 2005[104]	**0**	**0**	**+**		**0**	**0**	**+**		age, gender[s]
Schröder *et al*, 2004[106]		**0**				**0**			gender[s]
**Scandinavian region**									
Andersen *et al*, 2000[108]		**(+)**				**(+)**			gender[s]
Cubbin *et al*, 2006[112]				**+**[1], **+**[2]				**+**[1], **+**[2]	age, gender, urbanization, deprivation, marital status, immigration status, individual SEP
Engström, 2008[113]		**+**				**+**			age[†]

Author (Date)	Direction of the associations between the socioeconomic indicator and physical activity								
	Men				Women				Adjustment
	Income	Education	Social class	Other	Income	Education	Social class	Other	
Piro *et al*, 2007[138]	+	+		+[2]	+	+		+[2]	age, gender, marital status, education, employment status, income
Simonen *et al*, 2003[144]		0				0			-
**Anglo-Saxon region**									
Bartley *et al*, 2000[153] [c]			+				+		age
Ecob & Macintyre, 2000[156]				+[2]				+[2]	age, gender, social class, education, marital status, whether recently moved, household material deprivation
Livingstone *et al*, 2001[161]			0				+		gender[s]
Lunn, 2010[162]	+	+			+	+			gender, cohort, parent's participation in sports, education, income, occupation, occupation*gender
Popham & Mitchell, 2007[16]				+[1]				+[1]	age, gender[s], health status, employment status
Popham, 2010[169]			(+)				(+)		age, gender
Stamatakis & Chaudhury, 2008[170] [d]	+	+	+		+	+	+		-
Wagner *et al*, 2003[79] - IRE		0		0[3]					age, gender[s], marital status, obesity, reported disease
Wardle & Steptoe, 2003[172] [e]			+				+		age, gender, self-rated health

‡ The symbols in the table should be read as follows: '+' means that a high SEP is associated with a high VLTPA, a '-' means that a high SEP is associated with a low VLTPA. A '0' means that there was no association found. When the symbol is between brackets, no tests of significance were done.

† Reported for age group <45 and >45. They found the same association in both age samples.

a. Similar results found in the same sample population (employed subsample) by Schneider & Becker, 2005[76].
b. Similar results found in the same sample population by Scheerder *et al*, 2002[74].
c. The results reported here are from the sample from HALS 1984.
d. Similar results found in the same sample population by Poortinga, 2007[167] and Bartley *et al*, 2000[153] (sample HSfE 1993).
e. Similar results found in the same sample population by Wardle & Griffith, 2001[171].
s. Results were stratified according to gender.
t. All respondents were the same age.

1. Composite individual SEP measure
2. Neighbourhood SEP.
3. Household wealth (material conditions).

Table A5: Summary of study findings examining associations between <u>active transport (AT)</u> and SEP[‡]

Author (Date)	Direction of the associations between the socioeconomic indicator and physical activity								
	Men				Women				Adjustment
	Income	Education	Social class	Other	Income	Education	Social class	Other	
**EU wide studies**									
Van Tuyckom & Scheerder, 2010b[55]		+				+			-
**Western European region**									
van Lenthe *et al*, 2005[47]				-[2]				-[2]	age, gender, educational level
Van Dyck *et al*, 2010[23] – walking		+		-[2]		+		-[2]	age, gender, education, employment status, BMI, walkability of neighbourhood, neighb. SEP, walkability*neighb. SEP
Van Dyck *et al*, 2010[23] – cycling		+		-[2]		+		0[2]	age, gender, education, employment status, BMI, walkability of neighbourhood, neighb. SEP, walkability*neighb. SEP
Wagner *et al*, 2003[79] - FRA		0		-[3]					age, gender[s], marital status, obesity, reported disease, centre
**Eastern European region**									
Jurakić *et al*, 2009[81]	-	0			-	0			-
Kwaśniewska *et al*, 2010[28] – participation [b]	-	-			-	-			age, gender[s], education, income, marital status, smoking, place of residence, LTPA, OPA
Kwaśniewska *et al*, 2010[28] –minutes [b]	+	+			+	+			age, gender[s], education, income, marital status, smoking, place of residence, LTPA, OPA
**Scandinavian region**									
Andersen *et al*, 2000[108]		(-)				(-)			gender
Hu *et al*, 2003[37]		+				+			age, gender[s], study year
Mäkinen *et al*, 2009[125] [a]	0	0	+		+	0	0		age, gender[s], income, education, occupation
Wang *et al*, 2010[34]		+				+			age, gender[s], study year
**Anglo-Saxon region**									
Adams, 2010 – participation[29] [b]		0	0			0	0		age, gender, education, social class, employment status, car access
Adams, 2010 – minutes[29] [b]		+	-			+	-		age, gender, education, social class, employment status, car access
Wagner *et al*, 2003[79] - IRE		0		0[3]					age, gender[s], marital status, obesity, reported disease

‡ The symbols in the table should be read as follows: '+' means that a high SEP is associated with a high AT, a '-' means that a high SEP is associated with a low AT. A '0' means that there was no association found. When the symbol is between brackets, no tests of significance were done.

a. The results from the most recent data (1998-2002) are reported here.

b. The outcome was split up according to whether someone participates in active commuting and among those who participate, how many minutes they were active.

s. Results were stratified according to gender.

1. Composite individual SEP measure.
2. Neighbourhood SEP.
3. Household wealth (material conditions).

Table A6: Distribution of positive, negative, and null associations by SEP indicator and PA domain in different subsets of the reviewed associations.[a]

Physical activity[b,c]	Subset[d]	Total<br>TOTAL<br>n	+<br>%	0<br>%	-<br>%	Socioeconomic indicator<br>Income<br>n	+<br>%	0<br>%	-<br>%	Education<br>n	+<br>%	0<br>%	-<br>%	Social class<br>n	+<br>%	0<br>%	-<br>%	Other<br>n	+<br>%	0<br>%	-<br>%
**TPA**	**all**	**70**	**40%**	**27%**	**33%**	10	10%	70%	20%	32	44%	16%	41%	12	17%	25%	58%	16	69%	25%	6%
	**response**	**47**	**47%**	**19%**	**34%**	6	17%	50%	33%	21	48%	14%	38%	8	25%	13%	63%	12	75%	17%	8%
	**adjusted**	**46**	**37%**	**30%**	**33%**	4	0%	100%	0%	20	50%	15%	35%	12	17%	25%	58%	10	50%	40%	10%
	**sample size**	**54**	**50%**	**15%**	**35%**	4	25%	25%	50%	26	50%	15%	35%	10	20%	10%	70%	14	79%	14%	7%
**OPA**	**all**	**19**	**11%**	**26%**	**63%**	2	0%	100%	0%	8	25%	25%	50%	7	0%	14%	86%	2	0%	0%	100%
	**response**	**7**	**0%**	**0%**	**100%**	-	-	-	-	2	0%	0%	100%	3	0%	0%	100%	2	0%	0%	100%
	**adjusted**	**7**	**0%**	**0%**	**100%**	-	-	-	-	2	0%	0%	100%	3	0%	0%	100%	2	0%	0%	100%
	**sample size**	**13**	**15%**	**8%**	**77%**	-	-	-	-	4	50%	0%	50%	7	0%	14%	86%	2	0%	0%	100%
**TLTPA**	**all**	**200**	**68%**	**32%**	**1%**	34	59%	41%	0%	105	72%	27%	1%	38	74%	26%	0%	23	52%	48%	0%
	**response**	**147**	**70%**	**30%**	**0%**	26	58%	42%	0%	78	77%	23%	0%	28	71%	29%	0%	15	53%	47%	0%
	**adjusted**	**171**	**67%**	**33%**	**0%**	32	56%	44%	0%	83	75%	25%	0%	33	70%	30%	0%	23	52%	48%	0%
	**sample size**	**141**	**77%**	**23%**	**0%**	20	65%	35%	0%	72	85%	15%	0%	31	74%	26%	0%	18	61%	39%	0%
**VLTPA**	**all**	**110**	**76%**	**24%**	**0%**	24	75%	25%	0%	48	71%	29%	0%	20	85%	15%	0%	18	83%	17%	0%
	**response**	**82**	**77%**	**23%**	**0%**	18	78%	22%	0%	36	69%	31%	0%	16	88%	13%	0%	12	83%	17%	0%
	**adjusted**	**78**	**74%**	**26%**	**0%**	22	73%	27%	0%	28	68%	32%	0%	12	83%	17%	0%	16	81%	19%	0%
	**sample size**	**88**	**82%**	**18%**	**0%**	22	82%	17%	0%	32	78%	22%	0%	16	88%	13%	0%	18	83%	17%	0%
**AT**	**all**	**48**	**38%**	**29%**	**33%**	8	38%	13%	50%	26	54%	31%	15%	6	17%	50%	33%	8	0%	25%	75%
	**response**	**30**	**47%**	**17%**	**37%**	6	50%	17%	33%	16	63%	13%	25%	2	50%	50%	0%	6	0%	17%	83%
	**adjusted**	**40**	**40%**	**30%**	**30%**	6	50%	17%	33%	20	60%	30%	10%	6	17%	50%	33%	8	0%	25%	75%
	**sample size**	**36**	**39%**	**31%**	**31%**	6	50%	17%	33%	20	50%	30%	20%	6	17%	50%	33%	4	0%	25%	75%

[a] PA = physical activity.

[b] TPA = Total Physical Activity, OPA = Occupational Physical Activity, TLTPA = Total Leisure-time Physical Activity, VLTPA = Vigorous Leisure-time Physical Activity, AT = Active Transport.

[c] The symbol '+' indicates the positive associations (a high SEP is associated with a high PA), the symbol '-' indicates negative associations (a high SEP is associated with a low PA), and '0' indicates the neutral associations (no association found).

[d] All=Total set of studies and associations included in the review, Response=Subset of associations from studies with a reported response of 50% or higher, Adjusted=Subset of associations that were at least adjusted for age and gender, Sample size=Subset of associations from studies with a sample size of 2000 participants or higher.

Table A7: Distribution of positive, negative, and null associations by European region and PA domain in different subsets of the reviewed associations.[a]

		European Region																							
Physical activity[b, c]		EU wide studies				Western European region				Eastern European region				Southern European region				Scandinavian region				Anglo-Saxon region			
			+	0	-		+	0	-		+	0	-		+	0	-		+	0	-		+	0	-
	Subset[d]	n	%	%	%	n	%	%	%	n	%	%	%	n	%	%	%	n	%	%	%	n	%	%	%
**TPA**	**all**	2	100%	0%	0%	2	0%	100%	0%	10	40%	30%	30%	12	17%	8%	75%	20	40%	25%	35%	24	50%	33%	17%
	**response**	2	100%	0%	0%	-	-	-	-	6	67%	17%	17%	12	17%	8%	75%	17	47%	29%	24%	10	60%	20%	20%
	**adjusted**	2	100%	0%	0%	-	-	-	-	6	67%	17%	17%	6	0%	17%	83%	10	10%	40%	50%	22	45%	36%	18%
	**sample size**	2	100%	0%	0%	2	0%	100%	0%	6	67%	17%	17%	12	17%	8%	75%	14	50%	14%	36%	18	67%	11%	22%
**OPA**	**all**	2	100%	0%	0%	-	-	-	-	6	0%	67%	33%	-	-	-	-	5	0%	0%	100%	6	0%	17%	83%
	**response**	-	-	-	-	-	-	-	-	-	-	-	-	-	-	-	-	5	0%	0%	100%	2	0%	0%	100%
	**adjusted**	-	-	-	-	-	-	-	-	-	-	-	-	-	-	-	-	5	0%	0%	100%	2	0%	0%	100%
	**sample size**	2	100%	0%	0%	-	-	-	-	-	-	-	-	-	-	-	-	5	0%	0%	100%	6	0%	17%	83%
**TLTPA**	**all**	6	100%	0%	0%	37	81%	19%	0%	48	46%	52%	2%	28	71%	29%	0%	60	80%	20%	0%	21	48%	52%	0%
	**response**	-	-	-	-	32	84%	16%	0%	28	46%	54%	0%	18	83%	17%	0%	52	81%	19%	0%	17	35%	65%	0%
	**adjusted**	4	100%	0%	0%	34	79%	21%	0%	40	50%	50%	0%	24	75%	25%	0%	50	76%	24%	0%	19	42%	58%	0%
	**sample size**	6	100%	0%	0%	20	90%	10%	0%	24	54%	46%	0%	26	77%	23%	0%	47	87%	13%	0%	18	56%	44%	0%
**VLTPA**	**all**	-	-	-	-	28	96%	4%	0%	23	65%	35%	0%	19	37%	63%	0%	16	88%	13%	0%	24	88%	13%	0%
	**response**	-	-	-	-	22	95%	5%	0%	20	60%	40%	0%	14	42%	57%	0%	10	80%	20%	0%	16	100%	0%	0%
	**adjusted**	-	-	-	-	20	100%	0%	0%	18	56%	44%	0%	16	38%	63%	0%	10	100%	0%	0%	14	86%	14%	0%
	**sample size**	-	-	-	-	24	100%	0%	0%	19	58%	42%	0%	13	54%	46%	0%	12	100%	0%	0%	20	90%	10%	0%
**AT**	**all**	2	100%	0%	0%	12	33%	17%	50%	12	33%	17%	50%	-	-	-	-	12	50%	33%	17%	10	20%	60%	20%
	**response**	-	-	-	-	10	40%	10%	50%	8	50%	0%	50%	-	-	-	-	12	50%	33%	17%	-	-	-	-
	**adjusted**	-	-	-	-	12	33%	17%	50%	8	50%	0%	50%	-	-	-	-	10	60%	40%	0%	10	20%	60%	20%
	**sample size**	2	100%	0%	0%	4	0%	25%	75%	8	50%	0%	50%	-	-	-	-	12	50%	33%	17%	10	20%	60%	20%

[a] PA = physical activity.

[b] TPA = Total Physical Activity, OPA = Occupational Physical Activity, TLTPA = Total Leisure-time Physical Activity, VLTPA = Vigorous Leisure-time Physical Activity, AT = Active Transport.

[c] The symbol '+' indicates the positive associations (a high SEP is associated with a high PA), the symbol '-' indicates negative associations (a high SEP is associated with a low PA), and '0' indicates the neutral associations (no association found).

[d] All=Total set of studies and associations included in the review, Response=Subset of associations from studies with a reported response of 50% or higher, Adjusted=Subset of associations that were at least adjusted for age and gender, Sample size=Subset of associations from studies with a sample size of 2000 participants or higher.
